# Using a bladder syringe for retrograde urethrogram

**DOI:** 10.1308/rcsann.2025.0092

**Published:** 2025-12-15

**Authors:** R Karanjia, A Chetwood

**Affiliations:** Frimley Health NHS Foundation Trust, UK

## Background

Retrograde urethrograms are an essential diagnostic tool for any urologist. They are used both in elective settings to diagnose and delineate urethral stricture anatomy, but also in emergencies when urethral injury or disruption are suspected following pelvic trauma. Traditionally, a Foley catheter is placed within the fossa navicularis and the balloon partially inflated to tamponade the meatus before instilling contrast. However, the balloon inflation is often very painful for patients, and it is often technically difficult to inflate the balloon while keeping the catheter correctly positioned.

## Technique

**Figure 1:**
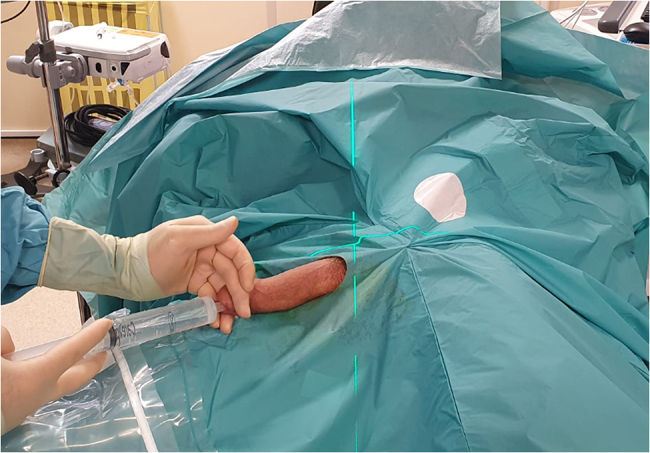
The tip of the bladder syringe is lubricated and placed inside the fossa navicularis. The tapered syringe, along with gentle pressure at the meatus, provides an excellent seal to perform a diagnostic urethrogram. Keeping the penis straight and use of the C-arm aiming beam minimises the risk of radiation exposure to the surgeon.

Instead of using a catheter, a 50ml bladder syringe can be filled with contrast. The tip of the syringe is lubricated with gel and inserted into the fossa navicularis. The natural tapering of the syringe tip, along with gentle finger pressure at the meatus, creates an excellent seal to perform a diagnostic urethrogram and is much less uncomfortable for the patient. Care should be taken by the operator to ensure their fingers are outside the imaged field to minimise radiation exposure. This can easily be navigated with adequate patient positioning, ensuring the penis is straight and with help from the C-arm aiming beam (Figure 1).

## Author contributions

**R Karanjia:** Conceptualization, Visualization, Writing – original draft. **A Chetwood:** Conceptualization, Writing – review & editing.

## Artificial intelligence

The author/s declare that no AI was used to conduct the study or prepare the manuscript.

